# Investigation of lacrimal gland and extraocular muscle in thyroid eye disease patients with severe subjective dry eye disease: a multiparametric magnetic resonance imaging study

**DOI:** 10.1007/s00417-024-06424-x

**Published:** 2024-03-08

**Authors:** Xulin Liao, Fatema Mohamed Ali Abdulla Aljufairi, Jake Uy Sebastian, Ruofan Jia, Hanson Yiu Man Wong, Kenneth Ka Hei Lai, Wanxue Chen, Zhichao Hu, Yingying Wei, Winnie Chiu Wing Chu, Clement Chee Yung Tham, Chi Pui Pang, Kelvin Kam Lung Chong

**Affiliations:** 1 Department of Ophthalmology and Visual Sciences, Faculty of Medicine, The Chinese University of Hong Kong, Hong Kong Eye Hospital, 147K Argyle Street, Hong Kong SAR, China; 2 Department of Ophthalmology, Salmaniya Medical Complex, Government Hospitals, Manama, 435 Bahrain; 3 Department of Statistics, The Chinese University of Hong Kong, Hong Kong SAR, China; 4 Department of Ophthalmology, The Prince of Wales Hospital, Hong Kong SAR, China; 5 Department of Imaging and Interventional Radiology, Faculty of Medicine, The Prince of Wales Hospital, The Chinese University of Hong Kong, Hong Kong SAR, China

**Keywords:** Thyroid Eye Disease, Dry Eye Disease, Lacrimal Gland, Extraocular Muscle, MRI

## Abstract

**Purpose:**

To analyze the radiological features of the lacrimal gland (LG) and extraocular muscle (EOM) in thyroid eye disease (TED) patients with severe subjective dry eye disease (DED) using magnetic resonance imaging (MRI) measurements.

**Methods:**

In this cross-sectional study, mechanical ocular exposure, dry eye assessment and MRI data were collected. Patients were classified into non-severe subjective DED group with ocular surface disease index (OSDI) < 33 and severe subjective DED group with OSDI ≥ 33. Linear regression model was applied for comparing the OSDI < 33 and OSDI ≥ 33 group in TED patients. The predictive performance of MRI parameters and models was assessed by receiver operating characteristic curve (ROC) analysis.

**Results:**

Consecutive 88 TED patients (176 eyes) were included in this study. In the OSDI < 33 group, 52 TED patients (104 eyes) with a mean clinical activity score (CAS) of 0.63 ± 0.75. In the OSDI ≥ 33 group, there are 36 TED patients (72 eyes), with a mean CAS of 1.50 ± 1.54. The age and sex of the patients were matched between the two groups. The OSDI ≥ 33 group had shorter tear break-up time, larger levator palpebrae superioris / superior rectus (LPS/SR), inferior rectus and lateral rectus, smaller LG, more inflammatory LPS/SR and inferior rectus than OSDI < 33 DED group (*P* < 0.05). In the linear regression analysis, compare to the OSDI < 33 DED group, the OSDI ≥ 33 group had larger medial rectus cross-sectional area (β = 0.06, 95%CI: (0.02, 0.10), *P* = 0.008), larger inferior rectus cross-sectional area (β = 0.06, 95%CI: (0.00, 0.12), *P* = 0.048), smaller LG cross-sectional area (β = -0.14, 95%CI: (-0.25, -0.04), *P* = 0.008). In the ROC analysis, the area under curve of medial rectus, inferior rectus, LG, and combined model are 0.625, 0.640, 0.661 and 0.716, respectively.

**Conclusion:**

Multiparametric MRI parameters of the LG and EOM in TED patients with severe subjective DED were significantly altered. Novel models combining the cross-sectional area of LG, medial rectus and inferior rectus showed good predictive performance in TED patients with severe subjective DED.


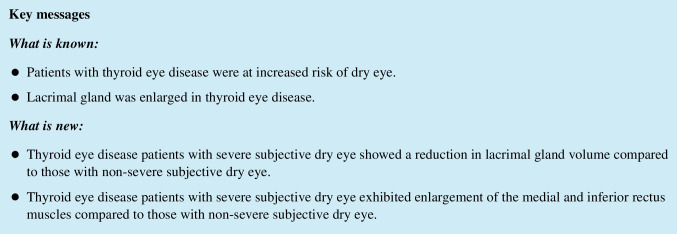


## Introduction

Thyroid eye disease (TED), also known as Graves' ophthalmopathy, Graves' orbitopathy, or thyroid associated orbitopathy, is a condition that affects the eye muscles and surrounding tissues [1]. It is commonly associated with hyperthyroidism caused by Graves' disease, although it can also occur in people with normal thyroid function or an underactive thyroid gland. TED is characterized by a range of symptoms, including vision loss, proptosis, eye pain, double vision, cosmetic changes, and dry eye symptoms [2, 3]. The underlying cause of TED is not fully understood, but it is thought to involve an immune response that causes inflammation and swelling of the tissues around the eyes, including the orbital fat, extraocular muscle (EOM), and lacrimal gland (LG) [4].

Dry eye disease (DED) is a complex disease of the ocular surface characterized by a loss of tear film homeostasis and accompanied by ocular symptoms, according to the definition of the Tear Film & Ocular Surface Society Dry Eye Workshop II (TFOS DEW II) [5]. This condition can be caused by various factors, including hormonal changes, environmental factors, and certain medications, and can present as aqueous-deficient, evaporative, mucin-deficient, or mixed type [5, 6]. DED in TED can be especially severe and significantly impact a patient's quality of life [7]. Therefore, early diagnosis of the underlying causes of DED in TED is crucial to develop effective treatments to alleviate this symptom. Regarding the research on DED in TED, there is a comparatively greater emphasis on investigating meibomian gland dysfunction (MGD) [8], while research on LG remains limited.

Located above the outer corner of each eye, the LG is responsible for producing the watery component of the tear film, which is crucial in maintaining the ocular surface and protecting the eye from infections and irritants [9]. Most studies show that TED's LGs are enlarged compared to healthy controls [10, 11]. Magnetic Resonance Imaging (MRI) is an imaging technique that can provide valuable information about the size, shape, and location of the LG, as well as any abnormalities or lesions that may be present in the surrounding structures.

The aim of this study is to examine changes of LG and EOM in TED patients with severe subjective DED, using radiological features derived from MRI measurements. The study also seeks to explore the potential clinical applications of novel multi-parametric MRI models for predictive DED in TED.

## Methods

### Study design and subjects

This is a cross-sectional study. The TED patients were recruited from the Chinese University of Hong Kong Medical Centre and the Chinese University of Hong Kong Eye Centre from September 2020 to December 2022. This study adhered to the tenets of the Declaration of Helsinki and Ethics approvals (KC/KE-10–0218/ER-3, NTEC Ref. 2010.594) obtained from the Chinese University of Hong Kong. The inclusion criteria for this study were included diagnosed as TED patients [2] and had not received any prior treatment for the condition, such as orbital radiation therapy or steroid pulse and more than 18 years old. However, patients who had incomplete clinical data, a history of refractive or other ocular surgery, ocular trauma, or Sjogren's syndrome were excluded from the study.

### Thyroid eye assessment

Margin reflex distance to the upper eyelids (MRD1) and lower eyelids (MRD2) were determined by measuring the distance between the eyelid margins and the center of the pupillary light reflex [12]. Exophthalmos was measured with a Hertel exophthalmometer [13] (Keeler Instruments Inc., Broomall, PA, USA), while lagophthalmos was measured by determining the distance between the upper and lower eyelid margins when the patient's eyes were closed [14]. Lateral flare was measured as the distance between the upper and lower eyelids on the lateral side of the corneal limbus [15]. A senior oculoplastic subspecialist conducted all the examinations. Each patient's clinical activity of TAO was assessed using the clinical activity score (CAS) recommended by the European Group on Graves' Orbitopathy, with a maximum score of 7 points.

### Dry eye assessment

We utilized the Ocular Surface Disease Index (OSDI) [16], which consists of 12 items and has a total score of 100. An OSDI score of 33 or higher indicated a severe level of subjective dry eye. Various measures were taken using the OCULUS Keratograph 5 M [17] (Oculus Optikgerate, Wetzer, Germany) including tear meniscus height (TMH), first and average non-invasive Keratography tear break-up time (NIKBUT first, NIKBUT average), and the Schirmer's test without anesthesia to assess aqueous tear production. Lipiview Interferometer [18] (TearScience Inc., Morrisville, NC) was used to measure the average, maximum, and minimum lipid layer thickness (LLT average, LLT maximum, LLT minimum), meibography, and blinking times. Meibography was graded using the meiboscore [19] system (0–3 score for one eyelid), where a score of 1 was considered mild, 2 was moderate, and 3 was severe meibomian gland dysfunction.

### MRI acquisition and processing

MRI was performed on a 3.0 T Siemens scanner (MAGNETOM Prisma, Siemens); using a 64-Channel Head/Neck coil [20]. All patients underwent T1-weighted images and T2-Short tau inversion recovery (STIR). T1-weighted imaging was carried out using turbo spin echo (TSE) technique at coronal plane: repetition time (TR) / echo time (TE) = 585/16 ms, acceleration factor for phase-encoding (Accel. factor PE) = 3, voxel size = 0.2 × 0.2 mm, matrix = 384 × 307, slice thickness = 3.0 mm, slice number = 26, flip angle = 130^◦^ number of averages = 3. T2-STIR imaging acquired with TSE technique at coronal and axial plane: TR/TE = 3300/60 ms, inversion time (TI) = 230 ms, turbo factor = 15, Accel. factor PE = 3, voxel size = 0.3 × 0.3 mm, matrix = 320 × 256, slice thickness/gap = 3.0 mm/0 mm, slice number = 26, flip angle = 160^◦^, number of averages = 2. Image analysis was performed by two oculoplastic fellows who were blinded to the clinical findings. The largest cross-sectional area of the EOM and the LG for both sides were manually traced with a dedicated workstation (Syngo. Via, Siemens, Erlangen, Germany) and measured three times on T1-weighted coronal images. The mean value of the 3 readings was used in statistical analysis. On T2-weighted coronal STIR sequences, the sections with the brightest signal of the muscle were identified three times. The same freehand tool was used to manually trace the region of interest, repeated 3 times and the mean value was recorded as activity. The signal intensity ratio (SIR) (rectus STIR / ipsilateral temporalis STIR) was calculated individually.

### Statistical analysis

The continuous variables were presented as mean ± standard deviation. The binary variables were expressed as percentage. The two-group comparison used the student t-test for continuous variables and the Chi-square test for categorical variables. The linear regression models were used to examine the TED patients in the OSDI < 33 and OSDI ≥ 33 groups, using MRI measurements as dependent variables and the OSDI category as an independent variable. The generalized estimating equation was used to adjust the inter-correlation between two eyes from the same subject. The receiver operating characteristic curve (ROC) curve was used to analyze the cross-sectional area of EOM between the eyes with or without severe subjective DED in TED patient. P value less than 0.05 was considered statistically significant. All the statistical analyses were performed using SPSS (IBM SPSS 23.0, SPSS Inc. Armonk, NY, USA) and R-Software R Project https://www.r-project.org/.

## Results

Out of the 88 patients with TED, a total of 36 patients (72 eyes) had an OSDI ≥ 33, while 52 patients (104 eyes) had an OSDI < 33. Both groups were matched for age and sex, with the OSDI ≥ 33 group having a mean age of 47.61 ± 10.96 years and 80.56% females, and the OSDI < 33 group having a mean age of 42.44 ± 13.49 years and 86.54% females. The CAS was significantly higher in the OSDI ≥ 33 group (1.50 ± 1.54) than in the OSDI < 33 group (0.63 ± 0.75), with *P* < 0.001. However, other parameters such as smoking status, Free Triiodothyronine (FT3), Free Thyroxine (FT4), Thyroid Stimulating Hormone (TSH), and Thyroid Stimulating Immunoglobulin (TSI) did not show any significant differences between the two groups (Table 1).

**Table 1:** Demographic Characteristics in 88 Thyroid Eye Disease Patients

	OSDI < 33	OSDI ≥ 33	*P*-value
Patient numbers	52	36	
Age(years)	42.44 ± 13.49	47.61 ± 10.96	0.060
Female	45 (86.54%)	29 (80.56%)	0.451
Smoker	8 (15.38%)	9 (25.00%)	0.261
FT3 (pmol/L)	6.08 ± 5.53	5.19 ± 2.26	0.324
FT4 (pmol/L)	18.98 ± 20.08	14.97 ± 7.03	0.132
TSH (mIU/L)	1.36 ± 1.85	1.40 ± 1.55	0.890
TSI	255.65 ± 159.72	302.09 ± 116.42	0.054
CAS	0.63 ± 0.75	1.50 ± 1.54	< 0.001

*FT3* Free Triiodothyronine, *FT4* Free Thyroxine, *TSH* Thyroid Stimulating Hormone, *TSI* Thyroid Stimulating Immunoglobulin, *CAS* clinical activity score, *OSDI* ocular surface disease index;

The study compared the mechanical ocular exposure and dry eye parameters between two groups of patients with different severity of subjective DED. The OSDI ≥ 33 group had significantly worse visual acuity (Log MAR) with a mean of 0.07 ± 0.14 compared to the OSDI < 33 group with a mean of 0.02 ± 0.10, with a *P* = 0.016. Additionally, the OSDI ≥ 33 group had a significantly shorter NIKBUT average with a mean of 14.30 ± 4.75 s compared to the OSDI < 33 group with a mean of 15.70 ± 4.42 s, with *P* = 0.047. However, other parameters such as IOP, MRD, lateral flare, lagophthalmos, exophthalmos, TMH, Schirmer’s test, NIKBUT first, LLT, blinking times, and meiboscore did not show significant differences between the OSDI < 33 and OSDI ≥ 33 group (Table 2, 3).

**Table 2:** Comparison of mechanical ocular exposure parameters of TED patients between OSDI < 33 and OSDI ≥ 33 group

	OSDI < 33	OSDI ≥ 33	*P*-value
Eye numbers	104	72	
Visual acuity (Log MAR)	0.02 ± 0.10	0.07 ± 0.14	0.016
IOP primary (mmHg)	15.62 ± 3.06	16.03 ± 2.89	0.364
IOP upgaze (mmHg)	24.41 ± 6.22	25.62 ± 6.64	0.229
MRD1 (mm)	5.36 ± 1.56	5.69 ± 1.65	0.157
MRD2 (mm)	5.12 ± 0.93	5.01 ± 1.01	0.531
Lateral flare (mm)	9.72 ± 1.96	9.81 ± 2.73	0.608
Lagophthalmos (mm)	0.53 ± 0.89	0.58 ± 1.02	0.942
Exophthalmos (mm)	18.32 ± 2.47	18.76 ± 3.14	0.544

*IOP* Intraocular Pressure, *MRD* Marginal Reflex Distance;

**Table 3:** Comparison of dry eye parameters of TED patients between OSDI < 33 and OSDI ≥ 33 group

	OSDI < 33	OSDI ≥ 33	*P*-value
Eye numbers	104	72	
TMH (mm)	0.31 ± 0.14	0.33 ± 0.15	0.341
Schirmer’s test (cm)	14.74 ± 10.39	12.49 ± 10.28	0.085
NIKBUT first (s)	9.94 ± 6.06	9.01 ± 5.12	0.288
NIKBUT average (s)	15.70 ± 4.42	14.30 ± 4.75	0.047
LLT average (nm)	71.42 ± 24.07	73.81 ± 21.14	0.499
LLT maximum (nm)	84.26 ± 20.15	87.62 ± 16.93	0.247
LLT minimum (nm)	56.79 ± 23.27	56.10 ± 23.16	0.846
Partial blinking	6.03 ± 5.91	4.82 ± 5.33	0.167
Total blinking	11.53 ± 5.82	10.69 ± 5.53	0.341
Meiboscore upper eyelid (0–3)	1.63 ± 0.90	1.85 ± 0.82	0.112
Meiboscore lower eyelid (0–3)	1.33 ± 0.60	1.42 ± 0.67	0.352

**Fig. 1:**
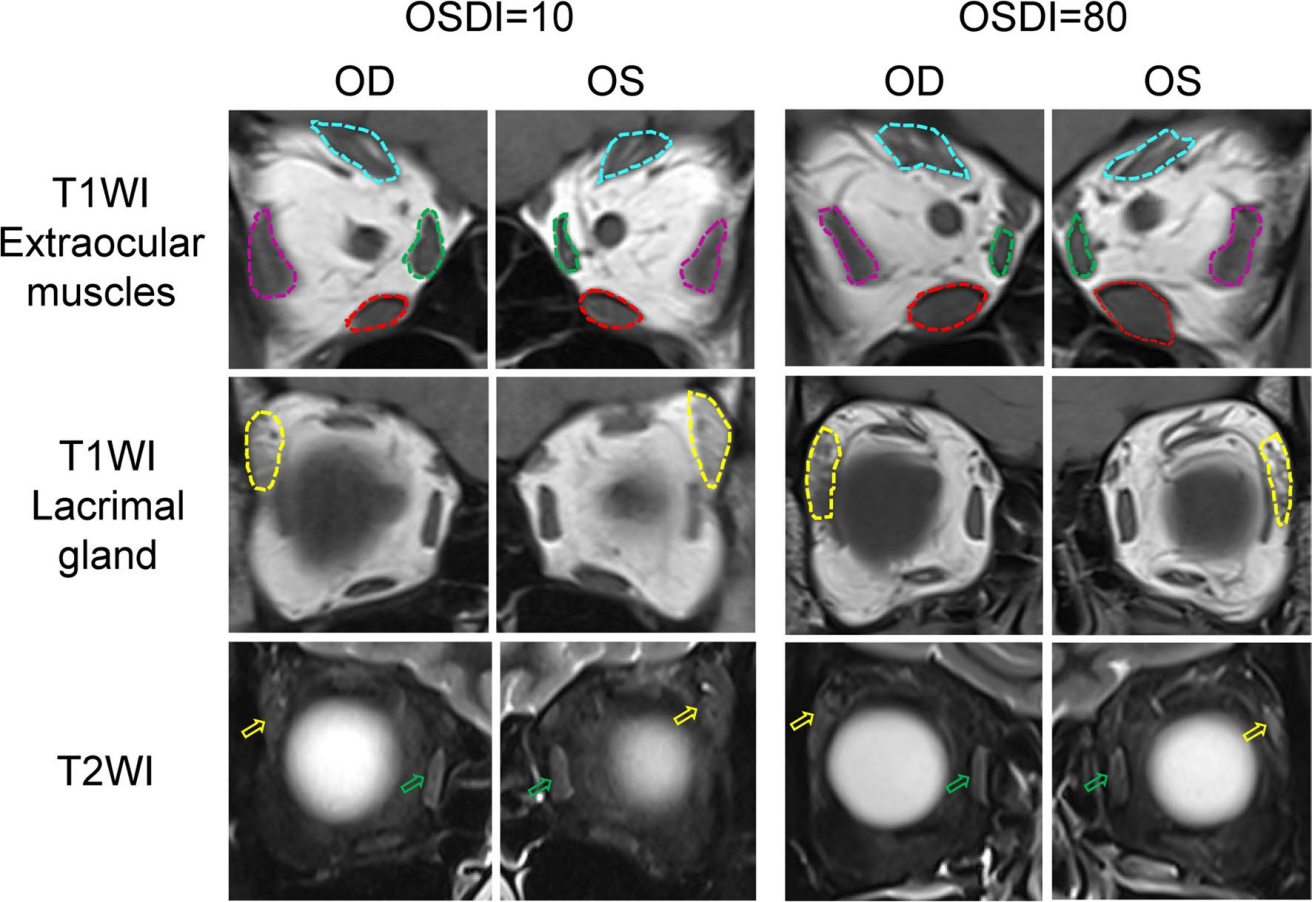
MRI scans of lacrimal gland and extraocular muscles. The blue dashed line represents the area of the superior rectus, the green dashed line represents the area of the medial rectus, the red dashed line represents the area of the inferior rectus, the purple line represents the lateral rectus, and the yellow dashed line represents the area of the lacrimal gland. The yellow arrow represents the lacrimal gland and the green one represents the medial rectus. **Abbreviation:** OSDI, Ocular Surface Disease Index

*TMH* Tear Meniscus Height, *NIKBUT* Non-Invasive Keratograph Break-Up Time, *LLT* Lipid payer thickness;

The OSDI ≥ 33 group showed larger cross-sectional areas of levator palpebrae superioris/superior rectus complex (LPS/SR) (0.40 ± 0.16 vs 0.34 ± 0.14 cm^2^), medial rectus (0.32 ± 0.13 vs. 0.26 ± 0.08 cm^2^), and inferior rectus (0.46 ± 0.17 vs. 0.40 ± 0.16 cm^2^) compared to the OSDI < 33 group, with *P* < 0.05. The SIR of LPS/SR (4.09 ± 1.46 vs. 3.61 ± 1.28) and inferior rectus (4.11 ± 1.43 vs. 3.68 ± 1.44) in OSDI ≥ 33 group were also larger than OSDI < 33 group, with *P* < 0.05. However, the OSDI ≥ 33 group had a smaller cross-sectional area of the LG (0.81 ± 0.26 vs. 0.96 ± 0.29 cm^2^) than the OSDI < 33 group (*P* < 0.05). There were no significant differences in the lateral rectus area or the SIR of medial rectus, lateral rectus, and LG between the two groups (Table 4, Fig. 1).

**Table 4:** Comparison of MRI quantitative measurements of TED patients between OSDI < 33 and OSDI ≥ 33 group

	OSDI < 33	OSDI ≥ 33	*P*-value
Eye numbers	104	72	
LPS/SR area (cm^2^)	0.34 ± 0.14	0.40 ± 0.16	0.008
Medial rectus area (cm^2^)	0.26 ± 0.08	0.32 ± 0.13	< 0.001
Inferior rectus area (cm^2^)	0.40 ± 0.16	0.46 ± 0.17	0.005
Lateral rectus area (cm^2^)	0.28 ± 0.17	0.30 ± 0.15	0.448
Lacrimal gland area (cm^2^)	0.96 ± 0.29	0.81 ± 0.26	< 0.001
LPS/SR SIR	3.61 ± 1.28	4.09 ± 1.46	0.011
Medial rectus SIR	3.96 ± 1.24	4.12 ± 1.35	0.288
Inferior rectus SIR	3.68 ± 1.44	4.11 ± 1.43	0.007
Lateral rectus SIR	3.49 ± 1.33	3.56 ± 1.13	0.491
Lacrimal gland SIR	3.24 ± 1.10	3.41 ± 0.95	0.106

*LPS/SR* Levator Palpebrae Superioris/Superior Rectus, *SIR* Signal Intensity Ratio.

**Fig. 2:**
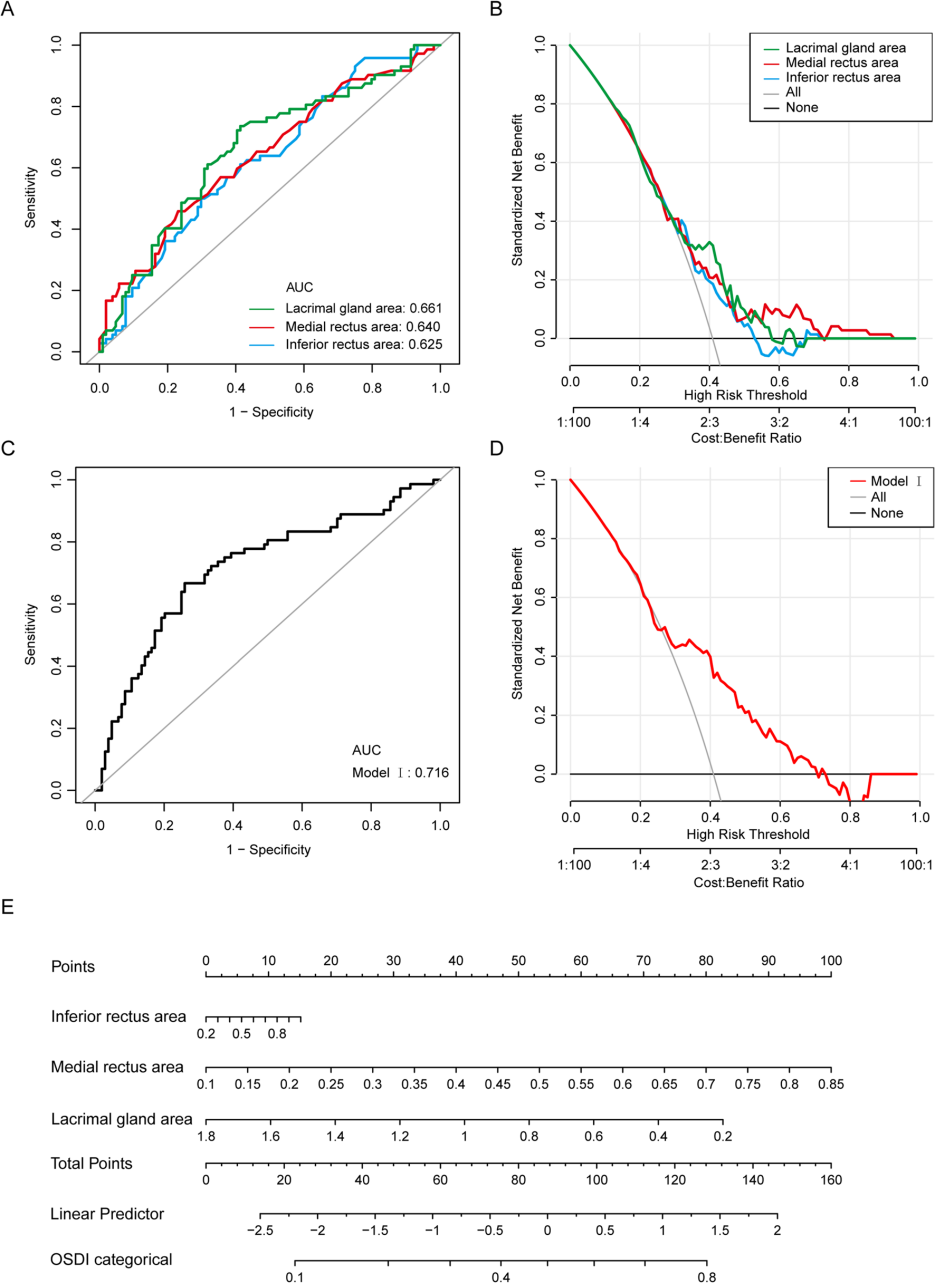
Receiver operating characteristic curves (ROC) and decision curve for evaluation of severe dry eye in thyroid eye disease (TED) patients. A. ROC of medial rectus area (red), inferior rectus area (blue) and Lacrimal gland area (green) for predicting severe dry eye in TED patients. B. Decision curve analysis of medial rectus area (red), inferior rectus area (blue) and Lacrimal gland area (green) for predicting severe dry eye in TED patients. C. ROC of model I for predicting severe dry eye in TED patients. D. Decision curve analysis of model I for predicting severe dry eye in TED patients. E. Nomogram of model I for predicting severe dry eye in TED patients

In the linear regression analysis, compare to the OSDI < 33 DED group, the OSDI ≥ 33 had larger medial rectus cross-sectional area (β = 0.06, 95%CI: (0.02, 0.10), *P* = 0.008), larger inferior rectus cross-sectional area (β = 0.06, 95%CI: (0.00, 0.12), *P* = 0.048), smaller LG cross-sectional area (β = -0.14, 95%CI: (-0.25, -0.04), *P* = 0.008) (Table 5). In the ROC analysis, the area under curve (AUC) of medial rectus, inferior rectus, LG, and combined model (inferior rectus area, medial rectus area and LG area) were 0.625, 0.640, 0.661 and 0.716, respectively (Table 6, Fig. 2).

**Table 5:** Linear regression analysis for comparing TED patients between OSDI < 33 and OSDI ≥ 33 group

	β	95%CI	*P*-value
LPS/SR area (cm^2^)	0.06	(-0.01, 0.12)	0.073
Medial rectus area (cm^2^)	0.06	(0.02, 0.10)	0.008
Inferior rectus area (cm^2^)	0.06	(0.00, 0.12)	0.048
Lateral rectus area (cm^2^)	0.02	(-0.05, 0.08)	0.572
Lacrimal gland area (cm^2^)	-0.14	(-0.25, -0.04)	0.008
LPS/SR SIR	0.48	(-0.07, 1.03)	0.090
Medial rectus SIR	0.16	(-0.38, 0.70)	0.558
Inferior rectus SIR	0.42	(-0.14, 0.99)	0.142
Lateral rectus SIR	0.07	(-0.38, 0.53)	0.748
Lacrimal gland SIR	0.17	(-0.20, 0.55)	0.367

*LPS/SR* Levator Palpebrae Superioris/Superior Rectus, *SIR* Signal Intensity Ratio (temporalis muscle Short Tau Inversion Recovery (STIR).

**Table 6:** Receiver operating characteristic curve analysis for severe subjective dry eye in TED patients

Variables	AUC	95%CI low	95%CI up	Best threshold	Specificity	Sensitivity
Medial rectus area (cm^2^)	0.625	0.542	0.708	0.435	0.702	0.500
Inferior rectus area (cm^2^)	0.640	0.556	0.724	0.295	0.769	0.458
Lacrimal gland area (cm^2^)	0.661	0.578	0.743	0.898	0.587	0.736
Model I	0.716	0.636	0.795	-0.431	0.740	0.667

*AUC* area under curve.

Predictive model:

Model I Logit (OSDI ≥ 33) = -0.39111 + 0.64388* inferior rectus area + 4.53123* medial rectus area -1.75653* lacrimal gland area

## Discussion

This is the first study presents novel findings on the use of MRI features for evaluating severe subjective DED in patients with TED. The investigation of LG and EOM revealed that severe subjective DED in TED patients was associated with reduced LG volume and enlarged medial and inferior rectus. The development of innovative models incorporating inferior rectus area, medial rectus area and LG area showcased good predictive performance in TED patients with severe subjective DED. The presence of the aforementioned MRI features could serve as a signal for ophthalmologists to focus on this aspect during clinical consultations with patients suffering from TED, enabling early treatment and advice to prevent complications.

There have been around a dozen clinical studies on the LG in TED (Table 7, 8). Among them, only the Ugradar’s study [21] investigated dry eye indicators, while most others [10, 22, 23] mainly indicated that the LG in more active TED is enlarged. Ugradar's study [21] concluded that Teprotumumab significantly reduces TED-related expansion of the LG, increases tear production, and improves dry eye symptoms. Our study results show that the LG of non-treatment TED patients with more severe subjective DED is smaller than those with non-severe subjective DED. Combined with previous studies results [10, 22, 23], TED severity correlates with larger LG (Table 8). Thus, we speculate that in patients with severe or active non-treatment TED, a less noticeable increase in gland size may suggest a risk of developing severe DED in the future. Combining our research with that of others shows that if CAS were positively associated with the LG, as the OSDI ≥ 33 group has a larger CAS, it implies that the severe subjective DED TED group experiences a greater degree of LG shrinkage, transitioning from a larger size (positively associated with CAS) to a smaller size (negatively associated with OSDI). Our findings have significant implications for early detection and treatment of DED in TED patients.

**Table 7:** Summary of clinical studies involved lacrimal gland in TED patients

Author	Year	Region	Study type	TED Patients numbers	Healthy Controls numbers
Ugradar et al. [21]	2023	USA	Prospective longitudinal	20	NA
Jiang et al. [10]	2022	China	Cross-sectional	28	14
Gao et al. [26]	2022	China	Retrospective	36	NA
Wu et al. [9]	2021	China	Cross-sectional	99	12 GD
Chen et al. [27]	2021	China	Cross-sectional	30	15
Hu et al. [28]	2020	China	Cross-sectional	47	NA
Gagliardo et al. [22]	2020	Italy	Cross-sectional	32	NA
Ishikawa et al. [23]	2019	Japan	Retrospective, observational case series	16	NA
Byun et al. [29]	2017	Korea	Cross-sectional	80	40
Hu et al. [30]	2016	China	Retrospective	33	24
Bingham et al. [11]	2014	USA	Retrospective	125	NA
Harris et al. [31]	2012	USA	Retrospective	128	NA

*TED* thyroid eye disease, *GD* Graves’ disease, *NA* not applicable.

**Table 8:** Summary of key conclusions of clinical studies involved lacrimal gland in TED patients

Author	Year	Key conclusions
Ugradar et al. [21]	2023	Teprotumumab significantly reduces TED related expansion of the LG, increases tear production, and improves dry eye symptoms
Jiang et al. [10]	2022	The combination of the T2-mapping value of LG and clinical indicators improved the stage prediction of TED compared to CAS
Gao et al. [26]	2022	LG prolapse measurements obtained from orbital MRI were positively correlated with CAS, proptosis and EOM volume
Wu et al. [9]	2021	Novel models combining LG T2 and ΔT1 values showed excellent predictive performances in diagnosing TED
Chen et al. [27]	2021	Readout-segmented echo-planar imaging-based diffusion tensor imaging is a useful tool to characterize the microstructural change of LG in patients with TED
Hu et al. [28]	2020	Structural MRI-based quantitative measurements at EOM, OF, and LG may serve as promising markers to predict response to glucocorticoid in patients with active and moderate–severe TED
Gagliardo et al. [22]	2020	Measurement of LG herniation seems to be a good marker of the disease and TED activity
Ishikawa et al. [23]	2019	Patients with TED who present with asymmetric LG enlargement need to be further evaluated
Byun et al. [29]	2017	The mean total EOM volume and LG volume was greater in active TED patients than other groups
Hu et al. [30]	2016	Quantitative measurements of the LG based on 3-T MR imaging may assist in the diagnosis and stage of TED
Bingham et al. [11]	2014	The LG is larger in patients with TED and correlates with subjective tearing and exophthalmometry
Harris et al. [31]	2012	LG is statistically significantly enlarged in TED

*TED* thyroid eye disease, *LG* lacrimal gland, *CAS* clinical activity score, *MRI* magnetic resonance imaging, *EOM* extraocular muscles, *OF* orbital fat, *SIR* signal density ratio.

We observed that patients with severe subjective DED in TED had larger medial rectus and inferior rectus muscles compared to those with mild to moderate subjective DED. We hypothesized that this finding may be related to the muscles' role in controlling eye movement. Specifically, an increase in the size or thickness of the medial rectus muscle, which controls inward eye movement, can affect horizontal eye movement and cause visual fatigue. In a study by Inoue et al. [24] on TED-related meibomian gland dysfunction, the authors also reported medial rectus enlargement, consistent with our findings, suggesting that this may be related to severe DED in TED patients. Furthermore, inferior rectus dysfunction can result in lagophthalmos [25] and increase corneal exposure. Lagophthalmos, which can lead to exposure of the eyeball, is also a contributing factor to subsequent dry eye in TED patients. Therefore, it is important to consider the potential implications of medial and inferior rectus muscle enlargement when assessing and treating patients with TED-related DED.

Our study has several limitations that need to be addressed. A large-scale, multicenter, and long-term randomized clinical trial is necessary to validate our hypotheses. Despite achieving an AUC of 0.716 in our combined model, there is room for further exploration to develop a more accurate model. Additionally, since the study used OSDI as the category, in future studies, we can focus on studying LG changes in specific dry eye subgroups, such as TED patients with severe MGD, or TED patients with severe corneal defects. Moreover, we need to further investigate the molecular mechanisms responsible for the severe subjective DED associated with LG shrinkage in TED patients. This will enable us to gain a deeper understanding of the disease and develop better treatment strategies.

In conclusion, the present study has demonstrated a significant association between the reduction in LG size and enlargement of the medial rectus and inferior rectus muscles in TED patients with severe subjective DED. Furthermore, the integration of these three factors into a predict model resulted in a high level of accuracy in the prediction of severe subjective DED in TED patients. These findings have important implications for early management of TED-related ocular surface disease.
